# Development and Validation of a Machine Learning Method Using Vocal Biomarkers for Identifying Frailty in Community-Dwelling Older Adults: Cross-Sectional Study

**DOI:** 10.2196/57298

**Published:** 2025-01-16

**Authors:** Taehwan Kim, Jung-Yeon Choi, Myung Jin Ko, Kwang-il Kim

**Affiliations:** 1 Silvia Health Inc., Seoul, Republic of Korea; 2 Department of Internal Medicine, Seoul National University College of Medicine, Seoul, Republic of Korea; 3 Department of Internal Medicine, Seoul National University Bundang Hospital, Gyeonggi-doSeongnam-si, Republic of Korea, 82 82-031-787-7032, 82 82-031-787-4052

**Keywords:** frailty, cross-sectional study, vocal biomarkers, older adults, artificial intelligence, machine learning, classification model, self-supervised

## Abstract

**Background:**

The two most commonly used methods to identify frailty are the frailty phenotype and the frailty index. However, both methods have limitations in clinical application. In addition, methods for measuring frailty have not yet been standardized.

**Objective:**

We aimed to develop and validate a classification model for predicting frailty status using vocal biomarkers in community-dwelling older adults, based on voice recordings obtained from the picture description task (PDT).

**Methods:**

We recruited 127 participants aged 50 years and older and collected clinical information through a short form of the Comprehensive Geriatric Assessment scale. Voice recordings were collected with a tablet device during the Korean version of the PDT, and we preprocessed audio data to remove background noise before feature extraction. Three artificial intelligence (AI) models were developed for identifying frailty status: SpeechAI (using speech data only), DemoAI (using demographic data only), and DemoSpeechAI (combining both data types).

**Results:**

Our models were trained and evaluated on the basis of 5-fold cross-validation for 127 participants and compared. The SpeechAI model, using deep learning–based acoustic features, outperformed in terms of accuracy and area under the receiver operating characteristic curve (AUC), 80.4% (95% CI 76.89%‐83.91%) and 0.89 (95% CI 0.86‐0.92), respectively, while the model using only demographics showed an accuracy of 67.96% (95% CI 67.63%‐68.29%) and an AUC of 0.74 (95% CI 0.73‐0.75). The SpeechAI model outperformed the model using only demographics significantly in AUC (*t*_4_=8.705 [2-sided]; *P*<.001). The DemoSpeechAI model, which combined demographics with deep learning–based acoustic features, showed superior performance (accuracy 85.6%, 95% CI 80.03%‐91.17% and AUC 0.93, 95% CI 0.89‐0.97), but there was no significant difference in AUC between the SpeechAI and DemoSpeechAI models (*t*_4_=1.057 [2-sided]; *P*=.35). Compared with models using traditional acoustic features from the openSMILE toolkit, the SpeechAI model demonstrated superior performance (AUC 0.89) over traditional methods (logistic regression: AUC 0.62; decision tree: AUC 0.57; random forest: AUC 0.66).

**Conclusions:**

Our findings demonstrate that vocal biomarkers derived from deep learning–based acoustic features can be effectively used to predict frailty status in community-dwelling older adults. The SpeechAI model showed promising accuracy and AUC, outperforming models based solely on demographic data or traditional acoustic features. Furthermore, while the combined DemoSpeechAI model showed slightly improved performance over the SpeechAI model, the difference was not statistically significant. These results suggest that speech-based AI models offer a noninvasive, scalable method for frailty detection, potentially streamlining assessments in clinical and community settings.

## Introduction

Global population aging is undergoing a profound transformation as the number of older adults continues to rise at an unprecedented rate. South Korea is undergoing an accelerated aging trend coupled with one of the world’s most dwindling birth rates. By 2025, South Korea is predicted to become a superaged society, with the proportion of people aged ≥65 years making up 20% of the population. This demographic shift is expected to continue, with estimates indicating that nearly 44% of South Korea’s population will be older than 65 years expected by 2050 [1].

Among the older population, frailty is a very common and significantly important geriatric condition because it affects health-related status, quality of life, place of residence, and mortality [2]. Frailty is a common geriatric syndrome characterized by a decline in physiological reserves and increased vulnerability to stressors. Early diagnosis and management of frailty are important not only for tailoring care plans and predicting adverse health outcomes at the individual level but also for strategizing public health initiatives that meet the distinct requirements of the rapidly growing older population [3].

Although the concept of frailty is universally recognized, methods for measuring frailty have not yet been standardized. The two most commonly used methods to identify frailty are the frailty phenotype and the frailty index [4,5]. However, these two methods have limitations in clinical application as they require measuring the frailty phenotype through a predetermined methodology or collecting various variables, making them not easily adaptable and usable in clinical settings. Another method for assessing frailty is the use of questionnaires [6]. As measuring frailty involves time, cost, and labor, it is necessary to develop a simplified, universally implantable, and convenient methodology to screen for frailty.

A recent study explored the integration of machine learning to enhance frailty detection, demonstrating that the choice of classifier and feature selection significantly impacted model performance, particularly when combining clinical and nonclinical data [7]. Voice biomarkers have been successfully used to identify acute diseases such as COVID-19, cognitive dysfunction, Parkinson disease, and psychiatric disorders [8-11]. Early research on detecting frailty using voice biomarkers often relied on simple methods, such as brief, predetermined vocal tasks [12]. Rosen-Lang et al [13] demonstrated the feasibility of using more sophisticated voice biomarkers for frailty classification, highlighting their potential for improving diagnostic accuracy in this population. Furthermore, the applicability of vocal biomarkers extends beyond frailty detection. Kaufman et al [14] demonstrated that acoustic analysis could effectively predict type 2 diabetes using voice segments, highlighting the versatility of voice biomarkers in noninvasive health diagnostics across various conditions.

The aim of this study was to develop and validate a classification model for predicting frailty status using vocal biomarkers in community-dwelling older people based on the voice recordings obtained from a picture description task (PDT) conducted for 2 minutes via a tablet and modeling with a machine learning algorithm.

## Methods

### Study Design

In this prospective cross-sectional study for developing and validating an artificial intelligence (AI) model to predict frailty status from vocal biomarkers, we recruited participants aged ≥50 years. We posted research promotion posters at Seoul National University Bundang Hospital to recruit participants. We enrolled those patients or their caregivers who expressed interest after seeing the posters and consented to participate in our study. Exclusion criteria were patients who (1) were diagnosed with dysarthria; (2) had a score of ≥10 on the Korean version of the Short Form Geriatric Depression Scale (SGDS-K) score of ≥10; (3) had a history of uncontrolled sleep disorders, anxiety, or behavioral disorders within 3 months; and (4) were deemed unable to comply with the study at the discretion of the researcher.

### Ethical Considerations

This study received ethics review and approval from the Institutional Review Board of Seoul National University Bundang Hospital (approval number B-2107-698-302) for the collection of data from participants in the clinical study. All data used in the study were collected and processed in accordance with the institutional review board guidelines. Informed consent was obtained from all participants for the collection of voice data. Participants were informed that their data would be anonymized or deidentified to protect their privacy and confidentiality.

### Data Collection

Voices were recorded in an academic tertiary hospital. The voice recording device, a Galaxy Tablet A7 LTE (SM-T505; Samsung Electronics Co, Ltd), was located between the participant and clinician at a distance of approximately 100 cm. The recordings were performed using an application developed for the PDT by Silvia Health, Inc. The recorded speech signals were digitized at a 48 kHz sampling rate. Audacity (version 3.3.3; Audacity Team) was used to remove noise other than participants’ voices.

### Development of the Korean Version of the PDT

We conducted experiments to estimate the frailty status from speech signals. We used the PDT, which is commonly used for screening cognitive function impairments, to collect speech data. In the PDT process, a picture is shown, and the participants are asked to describe everything visible and explainable in the picture for 2 minutes [15]. The participant’s cognitive function is assessed based on which part of the picture the participant observes and how they express the situations in syntactic and semantic terms. The PDT is usually used as a screening tool for Alzheimer disease, one of the types of degenerative brain disease, and we adopted the PDT as a tool to collect voice data for developing an AI model to evaluate frailty status [16,17].

The Boston Cookie Theft picture has been commonly used in the PDT to assess cognitive function; however, it does not fit South Korea’s culture [18]. For this reason, we developed a Korean version of the PDT, which contains Korean cultural references and could thus feel familiar to Koreans. The illustration was designed according to the following principles [19]: (1) salience of information, (2) semantic categories, (3) referential cohesion, (4) causal and temporal relations, (5) mental state language, (6) structural language, and (7) general cognition and perception. Detailed descriptions for each principle are represented in Multimedia Appendix 1.

The picture shown in Multimedia Appendix 2 was developed on the basis of these principles for the Korean version of the PDT. In our data acquisition phase, the picture was provided to the participants through an app developed for a tablet, and they were asked to describe it for 2 minutes. While they explained the picture, the participants’ voices were recorded using the application.

### Short Form of the Comprehensive Geriatric Assessment

The participants completed a short form of the Comprehensive Geriatric Assessment (SF-CGA) to identify comorbidities, functional status, cognitive function, and depressive symptoms. Medical history, including hypertension, diabetes, heart failure, atrial fibrillation, chronic kidney disease, chronic liver disease, respiratory disease, cerebrovascular disease, myocardial infection, Parkinson disease, dementia, and depression, was assessed. Cognitive function was assessed using the Korean version of the Mini-Mental State Examination-2 (K-MMSE-2) [20]. In addition, the Clinical Dementia Rating scale (CDR), a numerical scale used to quantify the severity of dementia symptoms, was assessed [21]. Functional status, including activities of daily living (ADLs) and instrumental ADLs (IADLs), were assessed using the Barthel index and the Lawton and Brody index, respectively [22,23]. Depressive symptoms of the participants were evaluated with the SGDS-K [24].

### Outcomes and Definition of Frailty and Prefrailty

Frailty status was defined using the Korean version of the Fatigue, Resistance, Ambulation, Illnesses, and Loss of Weight Scale (K-FRAIL) questionnaire [6]. We considered K-FRAIL scale scores with ≥3 positive items to indicate frailty. K-FRAIL scores of 1 or 2 were classified as prefrail, and a K-FRAIL score of 0 was categorized as robust. In this study, since the prefrail group is considered to exhibit some physiological deficits, the prefrail and frail groups were combined and compared with the robust group, which is consistent with previous research [25-27].

### Acoustic Features

Recent AI technologies have been used in speech recognition, generation, and analysis. However, few studies have used speech signals as biomarkers to evaluate frailty using machine learning techniques. In our experiments, we considered using voice recordings to evaluate the frailty. Specifically, we converted raw speech data into acoustic features expressed in the frequency domain for the input of an AI model. The spectral characteristics of speech signals can be expressed using mel-frequency cepstral coefficients (MFCCs) and are well suited for various deep-learning methodologies [28].

In preprocessing using MFCCs, the audio signals are transformed from the time domain to the frequency domain via a Fourier transform after quantization to time-windowing segments. Subsequently, the mel scale is applied to the spectrum to reflect the sensitivity of the frequency band. The first “n” coefficients are then gathered after transforming the mel spectrum through the discrete cosine transform. The gathered coefficients are called MFCCs and represent the unique characteristics of the audio signal. In general, studies using MFCCs have considered not only the coefficients but also their first- or second-order derivatives. In our study, to develop an AI model for identifying frailty using speech signals, we set the number of coefficients “n” to 20 and used the first- and second-order derivatives. Consequently, the acoustic features constructed using the MFCC technique comprised 60 dimensions. To convert the speech signals into acoustic features, we used Python 3.8 (Python Software Foundation) and the Librosa library (Python Software Foundation).

### Prediction Model Development and Validation

AI models using demographic and speech data were trained and validated to predict the frailty status. Among the machine learning methods, we trained the models using a supervised learning scheme, and the dataset collected from the prospective cohort was used for training and validation. The entire collected dataset was separated for training and validation purposes; there was no overlap between the training and validation datasets.

We explored and compared three types of machine learning models to predict frailty. The first model, SpeechAI, was constructed to predict the frailty status using only the acoustic features of speech signals. In the second model, DemoAI, demographic data were used to predict the frailty status, and in the third model, DemoSpeechAI, demographic data and acoustic features were combined to predict the frailty status. A total of 3 primary modules were designed to construct these models. One was designed as a classifier to identify frailty status from the data represented in the embedding space. The others were designed to represent demographics and speech data as embedding vectors.

The SpeechAI model was organized to predict frailty using acoustic features extracted from voice signals and was constructed with two modules: speech embedding and classifier. The Transformer structure was used in the speech-embedding module to express the acoustic features in the vector space [29]. The vector expressed via the Transformer model is passed to the classifier module, which consists of one hidden layer to predict the frailty status. The SpeechAI model shown in Figure S1 of Multimedia Appendix 3 had approximately 612,000 deep-learning network parameters to be trained. Among the parameters, approximately 96% (587,520/612,000) belonged to the Transformer architecture, and the classifier module had approximately 25,000 parameters.

The DemoAI model consists of a demographically embedded module that projects demographic information provided as a categorical type in an embedding space and a classifier module that predicts frailty based on the information expressed in the embedding space, as shown in Figure S2 of Multimedia Appendix 3. The demographic embedding module receives chronological age categorized into 10-year intervals and sex as the input among demographic information. They are projected onto 2D and 4D continuous embedding spaces, and the embedding vectors are concatenated to express a joint vector. The classifier module, which consists of one hidden layer, receives the joint vector expressed by the demographically embedded module, and serves as a binary classifier that distinguishes between frailty and robustness. The DemoAI model had approximately 26,000 entire network parameters for training.

The DemoSpeechAI model shown in Figure S3 of Multimedia Appendix 3 was constructed using demographics, speech-embedding modules, and a classifier module. The demographics and acoustic features were projected onto each embedding space through the embedding modules and then combined as a joint embedding vector. Subsequently, the joint embedding vector was passed to the classifier module to estimate frailty. The embedding modules used in the DemoSpeechAI model have the same structure as those used in the DemoAI and SpeechAI models. The parameters of the DemoSpeechAI model were approximately 621,000, consisting of approximately 34,000 for the classifier and 587,000 for the embedding module.

To build and train the deep-learning model, we used the PyTorch (version 2.0.1; PyTorch Team) library for Python, and an early stopping technique was used to prevent overfitting of the models in the training phase [30]. The dataset was split into five folds for 5-fold cross-validation; 4 sets were used for training, and 1 set was used to analyze the performance of the models. Each fold was divided into a balanced number of robust and frail labels.

### Pretraining Strategy Using Self-Supervised Learning

We designed and modularized three types of models to predict the frailty status of the participants using deep neural networks. Among the modules constructing each model, the speech-embedding module contained a significant number of parameters to be trained compared with the amount of data collected from participants, accounting for approximately 95% of the parameters in the SpeechAI and DemoSpeechAI models. To address the risk of overfitting due to the limited data, we used a self-supervised learning (SSL) strategy [31,32]. In the SSL strategy, an AI model is pretrained on a different task before being fine-tuned for the primary task. This involves pretraining the model on a different dataset, which helps establish a robust parameter foundation. As a result, there is no need to train the parameters from scratch, allowing effective training with a relatively small dataset. To implement the SSL technique, we used the AIHub dataset from the Open AI Dataset Project (AIHub) for public purposes [33]. The AIHub dataset includes voice recordings of 1002 Korean participants, which we used for pretraining the speech embedding module of the SpeechAI and DemoSpeechAI models. Consequently, only about 5% of the parameters in these models, specifically those in the classifier and demographics embedding modules, were trained using the dataset collected from our prospective cohort.

## Results

A total of 127 Korean participants aged ≥50 years were recruited to collect data at Seoul National University Bundang Hospital from June to December 2022. We acquired demographics, SF-CGA results, and voice recordings and developed an AI model for predicting frailty status. Among the participants, 43 out of 127 participants (34%) were female, the average number of years of education was 11.787 (SD 4.732) years, and the mean age was 69.2 (SD 10.9) years.

Among the 65 participants in the frail group (prefrail and frail), 6 out of 65 (9%) had dependent ADLs, and 7 out of 65 (11%) had dependent IADLs. The mean scores for the K-MMSE-2, standard total T-score, and CDR-Sum of Boxes were 25.874 (SD 4.53), 44.748 (SD 13.4), and 0.591 (SD 1.3), respectively. In the CDR, 87 out of 127 participants (68%) had 0 points, 37 out of 127 participants (29%) had 0.5 points, and 3 out of 127 participants (2%) had 1 point or higher. The mean SGDS-K scores were 2.945 (SD 2.89).

According to the K-FRAIL results, 65 out of 127 participants (51%) belonged to the frail group. The participants in the prefrail to frail group were older (73.4 vs 64.9 years; *P*<.001), had lower education levels (10.2 vs 13.5 years; *P*<.001), lower cognitive function (K-MMSE-2, 23.9 vs 27.9; *P*<.001), more depressive symptoms (SGDS-K, 4.1 vs 1.7; *P*<.001), and dependent ADL and IADL levels (Table 1).

**Table 1.** Clinical characteristics of the study population.

Characteristics			All (N=127)	Groups		*P* value
				Robust (n=62)	Prefrail and frail (n=65)	
**Sex, n (%)**						
	Female		43 (33.9)	17 (27.4)	26 (40)	.19
Age (years), mean (SD)			69.2 (10.95)	64.9 (9.26)	73.4 (10.84)	<.001
Education (years), mean (SD)			11.8 (4.73)	13.5 (3.33)	10.2 (5.28)	<.001
**K-MMSE-2**^a^ **(points), mean (SD)**						
	Total score		25.9 (4.53)	27.9 (2.07)	23.9 (5.31)	<.001
	Standardized T-score		44.7 (13.40)	49.5 (10.24)	40.3 (14.47)	<.001
SGDS-K^b^ (points), mean (SD)			2.95 (2.89)	1.7 (2.27)	4.1 (2.93)	<.001
ADL^c^ independency, n (%)			121 (95.3)	62 (100)	59 (90.8)	.04
IADL^d^ independency, n (%)			120 (90.5)	62 (100)	58 (89.2)	.02
**CDR**^e^, **n (%)**						.003
	0.0		87 (68.5)	51 (82.3)	36 (55.4)	
	0.5		37 (29.1)	11 (17.7)	26 (40)	
	≥1.0		3 (2.4)	0 (0)	3 (4.6)	
CDR-SOB^f^ (points), mean (SD)			0.59 (1.30)	0.15 (0.32)	1.02 (1.68)	<.001

a K-MMSE-2: Korean version of the Mini-Mental State Examination-2.

b SGDS-K: Korean version of the Short Form Geriatric Depression Scale.

c ADL: activities of daily living.

d IADL: instrumental activities of daily living.

e CDR: Clinical Dementia Rating scale.

f SOB: sum of boxes.

We explored and compared 3 AI models for predicting the frailty status using speech only (SpeechAI), demographics only (DemoAI), and a combination of speech and demographics (DemoSpeechAI). In this study, we used 5-fold cross-validation to train and evaluate our models using a dataset corresponding to 127 participants. The entire dataset was randomly divided into 5 subsets, with each fold containing approximately 20% of the total samples. Each fold was used once as the validation set, while the remaining 80% of the samples were combined to form the training set. This process was repeated 5 times so that each fold served as the validation set exactly once. Consequently, all data points were used for validation once, and the performance metrics reported are the average results from these 5 validation runs. This approach ensures that the model’s performance is robust and generalizable, avoiding overfitting and clearly defining the proportion of samples used in the validation phase. The accuracy, area under the receiver operating characteristic (ROC) curve (AUC), sensitivity, and specificity were used to analyze the performance of the model.

**Figure 1.**
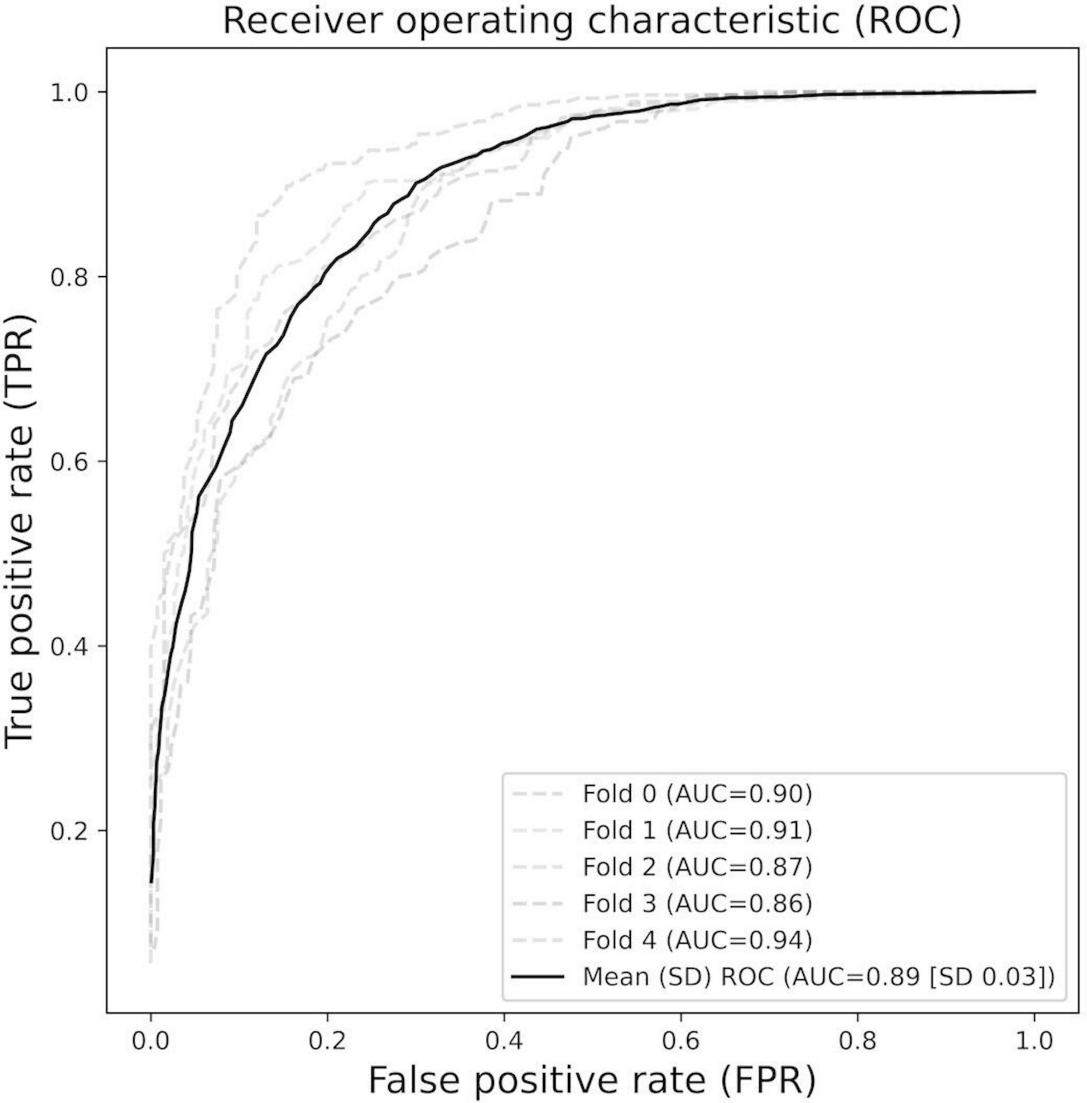
ROC curves for each fold demonstrating the performance of the SpeechAI model. AUC: area under the receiver operating characteristic curve; ROC: receiver operating characteristic.

The mean accuracy of the SpeechAI model for the 5 validation results was 80.4% (95% CI 76.89%‐83.91%), with minimum and maximum values of 75.05% and 86.93%, respectively. The mean AUC was 0.89 (95% CI 0.86‐0.92), with minimum and maximum values of 0.86 and 0.94, respectively. Furthermore, the average sensitivity and specificity of the SpeechAI model for 5-fold validation results were 0.75 (95% CI 0.67‐0.83) and 0.86 (95% CI 0.84‐0.88), respectively (Table 2). ROC curves were observed for 5-fold cross-validation, and the results and means are shown in Figure 1.

**Table 2.** The performance of the SpeechAI, DemoAI, and DemoSpeechAI models by 5-fold cross-validation.

		Accuracy (%)	AUC^a^	Sensitivity	Specificity
**SpeechAI**					
	Mean (95% CI)	80.40 (76.89‐83.91)	0.89 (0.86‐0.92)	0.75 (0.67‐0.83)	0.86 (0.84‐0.88)
	Minimum	75.05	0.86	0.62	0.83
	Maximum	86.93	0.94	0.88	0.88
**DemoAI**					
	Mean (95% CI)	67.96 (67.63‐68.29)	0.74 (0.73‐0.75)	0.65 (0.61‐0.69)	0.71 (0.68‐0.74)
	Minimum	67.51	0.73	0.60	0.66
	Maximum	68.44	0.75	0.71	0.75
**DemoSpeechAI**					
	Mean (95% CI)	85.60 (80.03‐91.17)	0.93 (0.89‐0.97)	0.89 (0.83‐0.95)	0.83 (0.77‐0.89)
	Minimum	74.31	0.83	0.75	0.73
	Maximum	92.29	0.98	0.95	0.91

a AUC: area under the receiver operating characteristic curve.

The curves represent the trade-off between true positive and false positive rates across different threshold values. The AUC values reflect the ability to distinguish between indviduals who were and were not frail.

**Figure 2.**
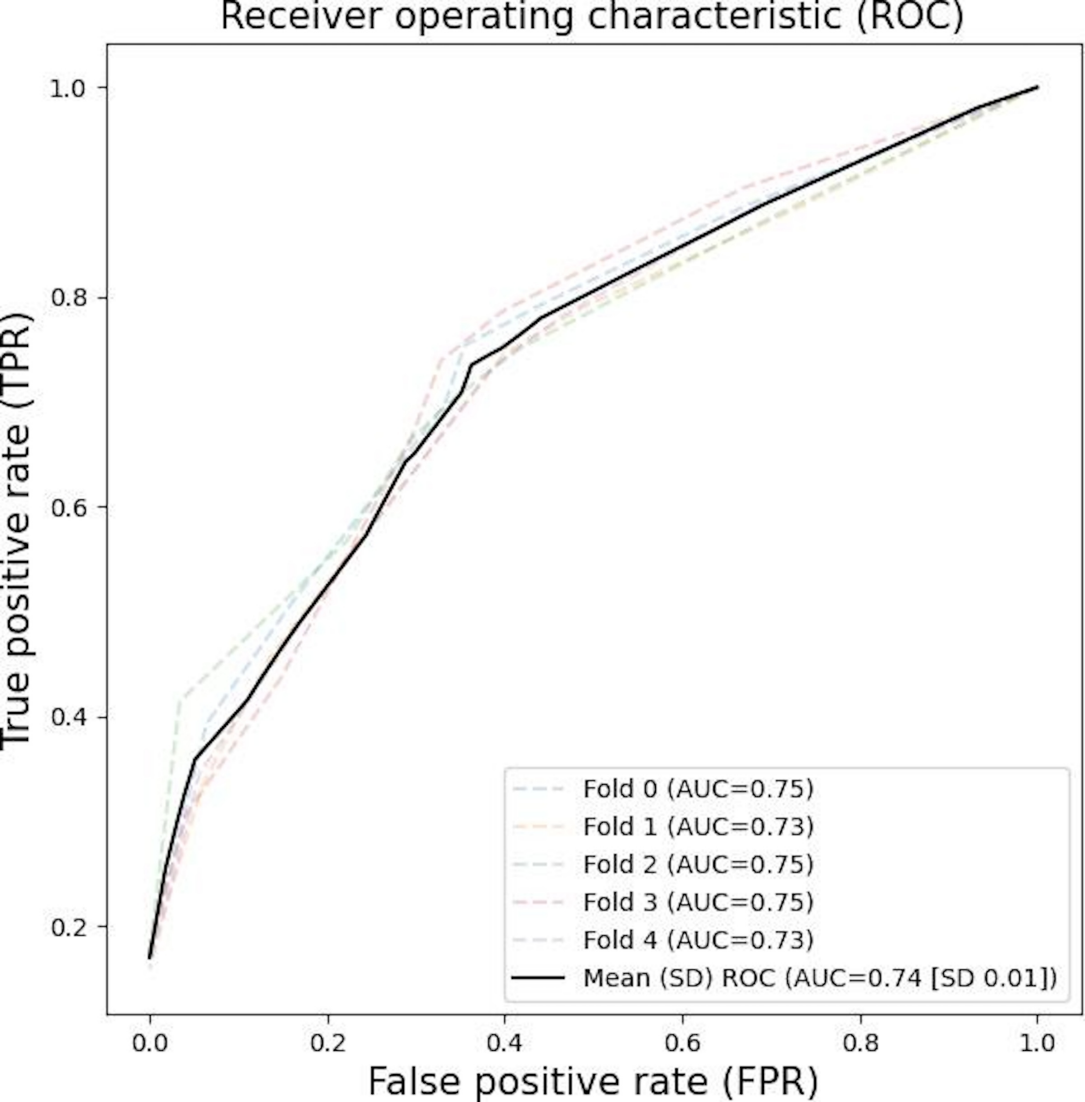
ROC curves for each fold demonstrating the performance of the DemoAI model. AUC: area under the receiver operating characteristic curve; ROC: receiver operating characteristic.

On the other hand, the performance of the DemoAI model using the validation dataset, which was equal to that used for the SpeechAI model validation, was 67.96% (95% CI 67.63%‐68.29%) for the mean accuracy, and its minimum and maximum values were 67.51% and 68.44%, respectively. The ROC curves of each fold by 5-fold cross-validation are shown in Figure 2, and the mean AUC was 0.74 (95% CI 0.73‐0.75), along with 0.73 and 0.75 for minimum and maximum values, respectively. Moreover, the mean sensitivity and specificity of the DemoAI model elicited from 5-fold cross-validation results were 0.65 (95% CI 0.61‐0.69) and 0.71 (95% CI 0.68‐0.74), respectively (Table 2).

The curves represent the trade-off between true positive and false positive rates across different threshold values. The AUC values reflect the ability to distinguish between individuals who were and were not frail.

**Figure 3.**
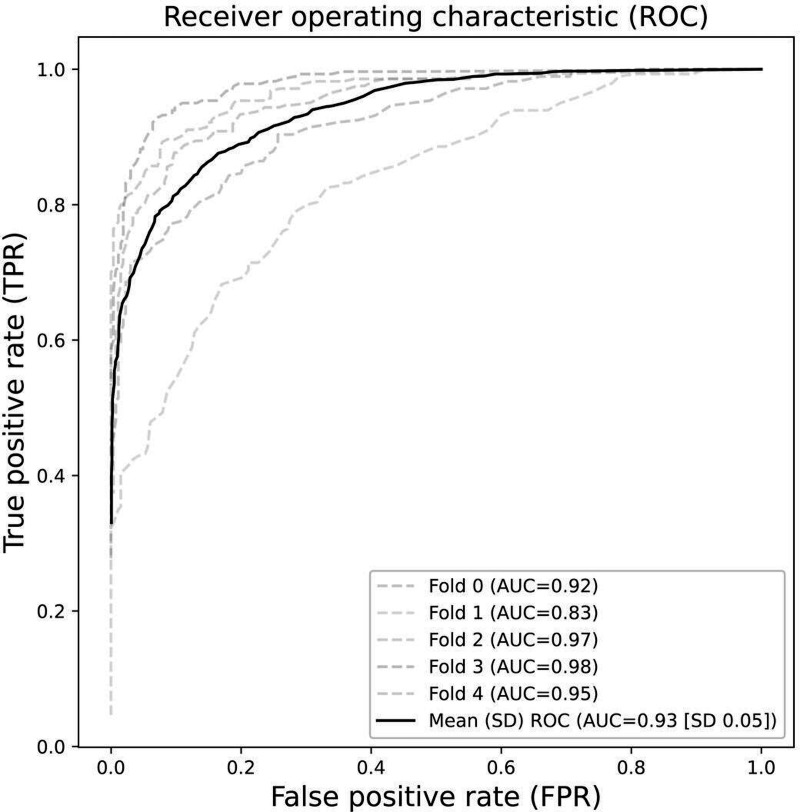
ROC curves for each fold demonstrating the performance of the DemoSpeechAI model. AUC: area under the receiver operating characteristic curve; ROC: receiver operating characteristic.

The mean accuracy of the DemoSpeechAI model for 5-fold cross-validation was 85.6% (SD 6.35%, 95% CI 80.03%‐91.17%), and the minimum and maximum values were 74.31% and 92.29%, respectively. Furthermore, the mean AUC values of the DemoSpeechAI model elicited from 5-fold cross-validation were 0.93 (SD 0.05, 95% CI 0.89‐0.97), and the mean sensitivity and specificity were 0.89 (SD 0.07, 95% CI 0.83‐0.95) and 0.83 (SD 0.07, 95% CI 0.77‐0.89), respectively (Table 2). Figure 3 shows the ROC curves obtained from 5-fold cross-validation and their mean.

In the comparison between the SpeechAI and DemoAI models, the SpeechAI (AUC 0.89, 95% CI 0.86‐0.92) model showed superior performance to the DemoAI model (AUC 0.74, 95% CI 0.73‐0.75) in AUC values (*t*_4_=8.705 [2-tailed]; *P*<.001), and the DemoSpeechAI model (AUC 0.93, 95% CI 0.89‐0.97) was superior to the DemoAI model in AUC values (*t*_4_=7.978 [2-tailed]; *P*<.001). However, there was no significant difference between the SpeechAI and DemoSpeechAI models (*t*_4_=1.057 [2-tailed]; *P*=.35).

To evaluate the effectiveness of our models using deep learning–based acoustic features, we conducted a comparative analysis with simpler machine learning models, specifically logistic regression, random forest, and decision tree. For this comparison, we used acoustic features extracted using the openSMILE toolkit [34], a commonly used tool for extracting audio features. The extracted features included frequency domain characteristics such as MFCCs, pitch, and loudness.

We trained logistic regression, random forest, and decision tree models using these acoustic features and compared their performance with our model, which use a transformer-based architecture for feature extraction. The results of this comparison are summarized in Table 3.

**Table 3.** Performance metrics for machine learning methods using acoustic features extracted by the openSMILE toolkit.

Model	Accuracy (%), mean (95% CI)	AUC^a^, mean (95% CI)	Sensitivity, mean (95% CI)	Specificity, mean (95% CI)
Logistic regression	61.45 (46.54‐76.35)	0.62 (0.47‐0.77)	0.62 (0.52‐0.71)	0.62 (0.36‐0.87)
Decision tree	57.48 (40.11‐74.84)	0.57 (0.40‐0.75)	0.59 (0.31‐0.86)	0.56 (0.41‐0.72)
Random forest	66.09 (57.69‐74.50)	0.66 (0.58‐0.75)	0.69 (0.58‐0.81)	0.63 (0.42‐0.84)

a AUC: area under the receiver operating characteristic curve.

The results in Table 3 compare with our model, SpeechAI, which uses the deep neural network–based feature extractor. SpeechAI significantly outperforms these traditional features-based models. The performance metrics for SpeechAI are as follows: accuracy: 80.40%, AUC: 0.89, sensitivity: 0.75, and specificity: 0.86. This demonstrates the superiority of our deep-learning approach, particularly in handling the complex and high-dimensional nature of acoustic features. The advanced feature extraction capabilities of the SpeechAI model enable it to capture intricate patterns and nuances in the voice data, which simpler models fail to exploit fully.

While traditional models provide a baseline performance, the deep learning–based model’s ability to leverage large-scale pretraining and complex feature extraction processes leads to improved predictive accuracy. These results underscore the effectiveness of deep-learning techniques for this application and highlight their potential for broader adoption in related research fields.

## Discussion

### Principal Findings

In recent years, several studies have investigated the use of voice data to predict frailty among older adults. One study explored the application of vocal biomarkers for frailty classification, finding significant associations between specific voice parameters and frailty levels [12]. Our study builds on this by using advanced deep-learning techniques to extract more nuanced features from voice data, potentially improving the accuracy and reliability of frailty predictions. In addition, a study demonstrated the feasibility of using voice biomarkers to classify frailty, focusing on various acoustic parameters such as formant frequencies and spectral energy ratios [13]. Our findings align with these results, further validating the effectiveness of voice biomarkers in predicting frailty. However, our approach differs by incorporating a more extensive set of acoustic features and using robust cross-validation methods to ensure generalizability. Our study aligns with the growing body of research that leverages vocal biomarkers to predict cognitive and functional decline. Notably, a study demonstrated the effectiveness of using spontaneous speech analysis to identify cognitive function decline among older adults in a multilanguage cross-sectional study [35]. Their findings highlighted that specific acoustic features could discriminate between healthy individuals and those with mild to severe cognitive impairment with high accuracy, supporting the viability of voice as a noninvasive diagnostic tool.

Using acoustic features to predict frailty is promising, particularly in the context we have adopted, where participants describe and explain a picture. Recent studies have demonstrated that transformer models yield superior results in various speech-related tasks, such as speech recognition, speech synthesis, and speech classification, surpassing traditional methods. Transformer architectures significantly improve the ability to capture complex patterns and long-range dependencies in sequential data, which are crucial for accurate speech processing [36].

In our study, we compared the performance of our transformer-based SpeechAI model with machine learning models that use acoustic features extracted by the openSMILE toolkit, which does not rely on deep neural networks. The openSMILE toolkit is widely used for extracting features such as pitch, loudness, and MFCCs. Our experiments showed that the deep neural network–based model outperformed simpler models using acoustic features, demonstrating the effectiveness of the transformer architecture in extracting and leveraging high-level acoustic features for predicting frailty.

The ability of the transformer architecture to model long-range dependencies and process sequences in parallel contributes to its enhanced performance [37]. This capability allows the transformer-based SpeechAI model to capture intricate patterns and nuances in the voice data, which extractors that do not rely on deep neural networks fail to exploit fully. The abilities can be experimentally observed by the superior performance metrics of SpeechAI, such as higher accuracy and better sensitivity and specificity, compared with models based on traditional acoustic feature extraction methods.

Through this study, the picture, Silvia Train Station, was developed for the Korean version of the PDT. It was designed according to the appropriate principles, containing Korean cultural references that would feel familiar to Koreans. We recruited a prospective cohort that included various clinical, medical, and demographic information and voice data encompassing the frailty status of 127 individuals. We developed and validated a model to predict frailty using AI techniques in conjunction with voice data from a prospective cohort and public AIHub dataset. Our study showed that 3 modularized models can be used to predict the participants’ frailty status using deep neural networks, which showed excellent performance.

### Evaluation With Imbalanced Validation Set

In our study, we investigated whether speech data could be used to classify frailty and observed meaningful results. We conducted experiments to classify between robust individuals and a combined group of individuals with prefrailty and frailty. We trained the model using a balanced class distribution between the two groups to ensure effective learning and avoid bias toward the majority class. Balancing the dataset during training is crucial, as it prevents the model from favoring the majority class, leading to improved performance across both classes and enhancing the model’s generalization capability. However, recognizing that the real-world prevalence of frailty is approximately 20%, we conducted an additional sensitivity analysis with the SpeechAI model to better reflect real-world conditions by using a validation set where the frailty group constituted 20% of the data. The results showed an accuracy of 74.80% (95% CI 69.36%‐80.25%), an AUC of 0.79 (95% CI 0.71‐0.88), a sensitivity of 0.77 (95% CI 0.67‐0.87), and a specificity of 0.63 (95% CI 0.39‐0.88). These results indicate that the model maintained strong predictive accuracy with the imbalanced validation set, demonstrating its robustness. While this approach provides meaningful insights, future research should aim to include more diverse and representative cohorts to fully validate the model’s applicability to real-world scenarios.

### Strengths and Limitations

Our study had several strengths. First, we developed and validated a model that successfully estimated the frailty state by showing a picture and obtaining voice recordings that freely described the picture using a tablet. Previous methods to diagnose frailty consumed a lot of time, space, and manpower or required much effort to process existing medical data. According to the methodology used in this study, older adults can check their risk of frailty in a way that can be performed on their own without having to visit a hospital or research institution. Second, voice data predicted frailty well, and it was experimentally confirmed that the acoustic features extracted from the voice recordings played an important role in predicting frailty. Based on these results, we demonstrated the possibility of developing many acoustic feature–derived models that can identify many health-related characteristics of older people, such as cognitive function, certain disease status, morbidity, and mortality. Third, despite statistical challenges, the model was developed and validated using a limited dataset, providing effective performance, even with a small sample size. The model efficiently leveraged voice data, omitted frailty-related variables, and demonstrated robust predictive capability while avoiding unnecessary complexity.

Our study had several limitations. First, in research using voice data, there are inevitable limitations in noise processing. The data collected for this study contained various noises as they were collected in a routine hospital environment. To remove noise, it was necessary to remove sounds other than the participants’ voices. So, it is necessary to provide an automatic noise removal process using technologies such as noise canceling, speaker recognition, and voice activity detection in further studies. Second, in the PDT, which was performed in our study, cognitive function could be assessed by the participants’ understanding of the causal relationship or the importance of the PDT. However, during the PDT, the participants had some difficulty explaining the given picture for 2 minutes, which made it difficult to collect complete and diverse sound characteristics from the participants. Therefore, if the PDT can be developed to be more specialized and suitable for the older population and to extract acoustic features, the accuracy of AI technology will further increase. Third, the relatively small sample size may not fully represent the diversity of each group, and biases introduced during participant selection could have affected the generalizability of the results. In addition, the study involved only Korean participants, thus caution should be taken in generalizing the results to populations from different regions or ethnicities. Furthermore, there is a significant age difference between the robust and prefrail and frail groups in our cohort. Due to this existing significant difference in age between the two groups, the DemoAI model, which was trained using only age and sex, also showed relatively high performance. Nevertheless, the model using speech data demonstrated statistically superior performance compared with the DemoAI model, highlighting the effectiveness of incorporating vocal biomarkers in frailty prediction. Future work should include more diverse participants to address these limitations and ensure the broader applicability of the findings. In addition, developing advanced noise processing techniques to improve the clarity of voice data collected in natural environments is essential. Expanding the use of vocal biomarkers to predict various health conditions and integrating these models into mobile health apps will make health monitoring more accessible. This approach could lead to noninvasive, efficient, and widely applicable health assessment tools for older adults.

### Conclusions

We developed and evaluated 3 types of models, DemoAI, SpeechAI, and DemoSpeechAI, to predict participants’ frailty status using deep neural networks with SSL techniques based on voice recordings collected via a tablet during the PDT. This was successfully developed using voice data collected from the recruited participants, assisted by an independently released voice database (AIHub). The next possible step would include applying the model to electronic health records, which can assist in decision-making, additional perioperative evaluation, and supportive care to prevent adverse outcomes after surgery.

## Supplementary material

10.2196/57298 Multimedia Appendix 1 Detailed descriptions of principles to build the illustrations for the picture description task.

10.2196/57298 Multimedia Appendix 2 Silvia Train Station (Copyright 2022. Silvia Health Inc.), an image used in the Korean version of the PDT.

10.2196/57298 Multimedia Appendix 3 Architecture of the three artificial intelligence models used for predicting frailty.

## Abbreviations

ADL: activities of daily living
AI: artificial intelligence
AUC: area under the receiver operating characteristic curve
CDR: Clinical Dementia Rating scale
IADL: instrumental activities of daily living
K-FRAIL: Korean version of the Fatigue, Resistance, Ambulation, Illnesses, and Loss of Weight Scale
K-MMSE-2: Korean version of the Mini-Mental State Examination-2
MFCC: mel-frequency cepstral coefficient
PDT: picture description task
ROC: receiver operating characteristic
SF-CGA: short form of the Comprehensive Geriatric Assessment
SGDS-K: Korean version of the Short Form Geriatric Depression Scale
SSL: self-supervised learning
