# Supplementary material for: Development and Validation of a Machine Learning Method Using Vocal Biomarkers for Identifying Frailty in Community-Dwelling Older Adults: Cross-Sectional Study
Source: JMIR Med Inform. 2025 Jan 16;13:e57298. doi: 10.2196/57298 (PMC11756832; doi:10.2196/57298)
Supplement: Multimedia Appendix 1 [file medinform-v13-e57298-s001.pdf]

Detailed Descriptions of Principles to Build the Illustrations for the Picture Description Task (PDT)

(1) Salience of Information
The pictures were organized into high- and low-salience scenes. Participants without neurological impairment typically described the pictures in order from the most to least important information. To assess a participant's cognitive function, the placement of items with varying degrees of salience in the structure of the picture were considered.

(2) Semantic Categories
The context of a picture contained animate entities, inanimate entities, and actions that could be represented using a wide range of semantic categories. Participants could describe the context at semantic levels, from general to specific terms, and different levels of representation enabled clinicians to assess their cognitive function.

(3) Referential Cohesion
The participant should be able to use the pronoun to describe the entities already mentioned; this process is also called an anaphoric reference. To assess this referential cohesion ability, entities that are difficult to distinguish with unclear pronouns were represented in the context of the picture.

(4) Causal and Temporal Relations
The picture given to participants in the PDT was a static image, but had causal and temporal events; events represented in the picture were temporally or causally linked. The participants could deduce this, and clinicians could thus assess their reasoning.

(5) Mental State Language
The mental states of the participants could be assessed from the events and actions that appeared in the picture and were used to judge the cognitive processes related to language in the theory of the mind.

(6) Structural Language and Speech
The picture included a context for evaluating the ability of generation skills to articulate scenes via a structural sentence.

(7) General Cognition and Perception
Assessment elements of general cognitive and perceptual functions for recognizing and understanding scenes were considered during the organization stage.
