# Supplementary material for: Development and Validation of a Machine Learning Method Using Vocal Biomarkers for Identifying Frailty in Community-Dwelling Older Adults: Cross-Sectional Study
Source: JMIR Med Inform. 2025 Jan 16;13:e57298. doi: 10.2196/57298 (PMC11756832; doi:10.2196/57298)

Silvia Train Station (Copyright 2022. Silvia Health Inc.), an image used in the Korean version of the Picture Description Task (PDT). Participants described the image in detail for 2 minutes, and their speech was recorded to build a classifier for identifying frailty. Vocal biomarkers were extracted from the collected data to predict frailty in older adults.

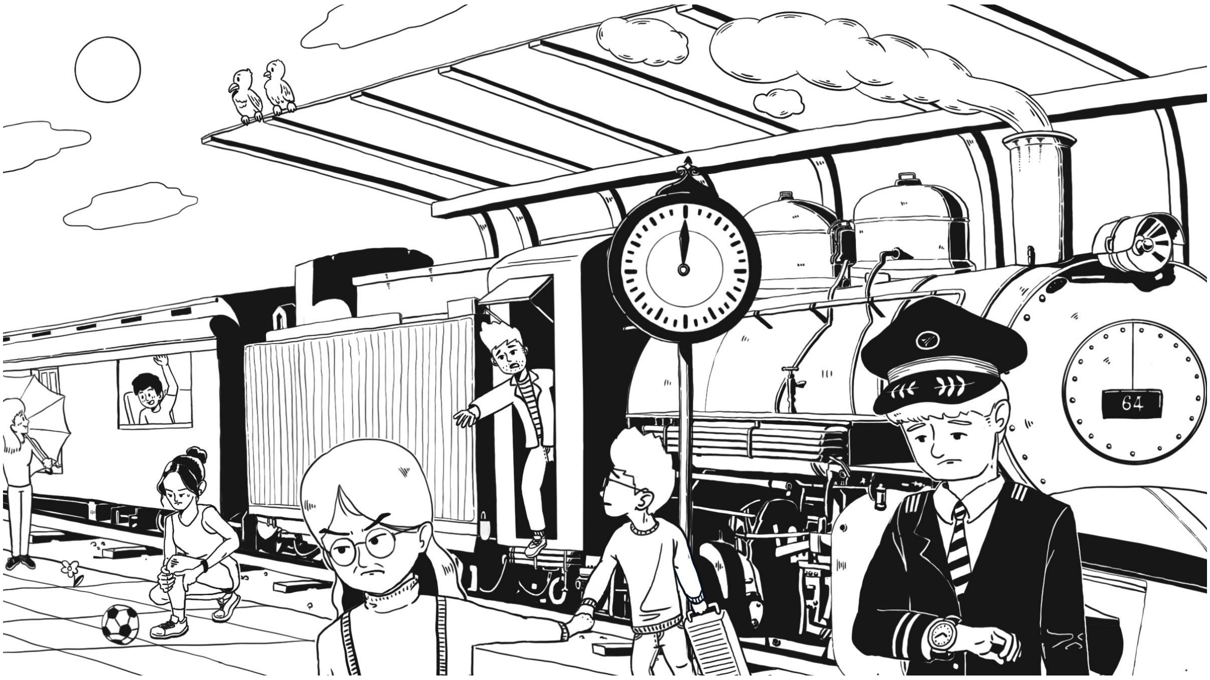

Supplement: Multimedia Appendix 2 [file medinform-v13-e57298-s002.pdf]
