# Supplementary material for: Development and Validation of a Machine Learning Method Using Vocal Biomarkers for Identifying Frailty in Community-Dwelling Older Adults: Cross-Sectional Study
Source: JMIR Med Inform. 2025 Jan 16;13:e57298. doi: 10.2196/57298 (PMC11756832; doi:10.2196/57298)
Supplement: Multimedia Appendix 3 [file medinform-v13-e57298-s003.pdf]

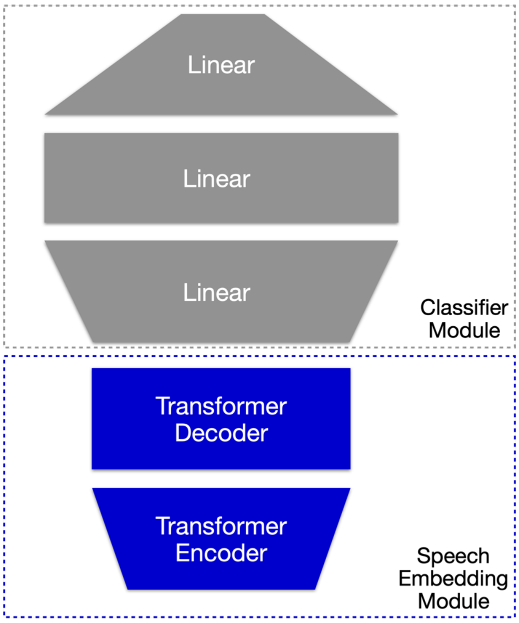

Figure 1S. Architecture of the SpeechAI model used for predicting frailty. The SpeechAI model utilizes deep-learning-based acoustic features extracted from voice recordings in the speech embedding module to predict frailty.

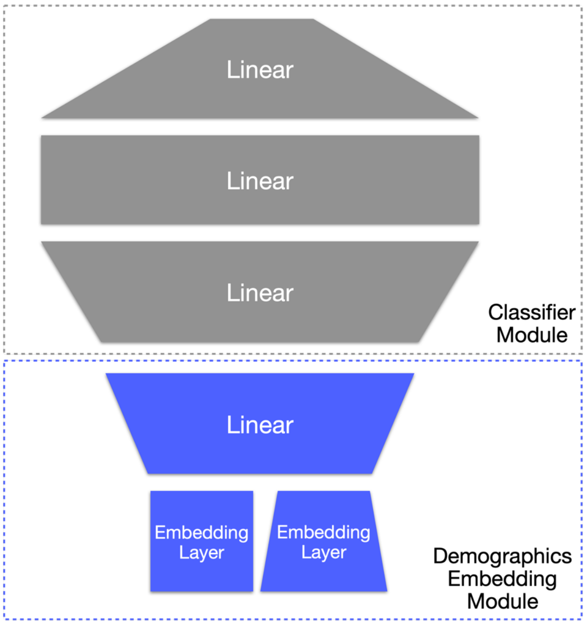

Figure 2S. Architecture of the DemoAI model used for predicting frailty. The DemoAI leverages demographic data only by embedding it into vector space in the demographics embedding module to classify frailty.

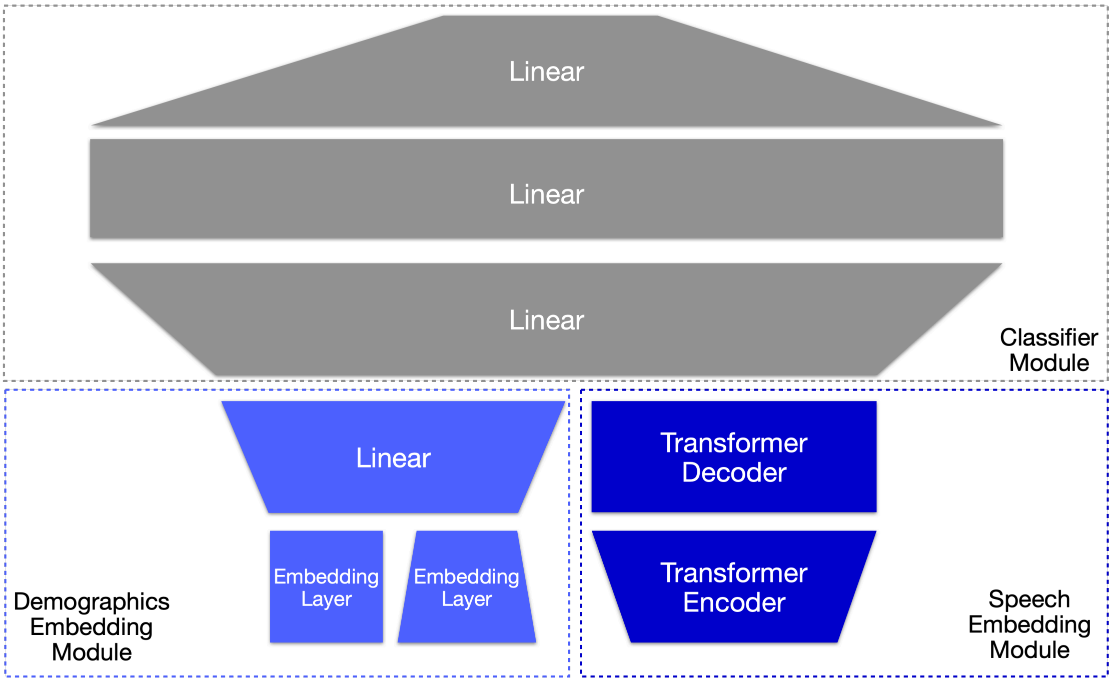

Figure 3S. Architecture of the DemoSpeechAI model used for predicting frailty. The DemoSpeechAI combines voice and demographic features represented through embedding modules to improve prediction accuracy.
